# Structural Control of Microvessel Diameters: Origins of Metabolic Signals

**DOI:** 10.3389/fphys.2017.00813

**Published:** 2017-10-24

**Authors:** Bettina Reglin, Timothy W. Secomb, Axel R. Pries

**Affiliations:** ^1^Department of Physiology and Center for Cardiovascular Research, Charité Universitätsmedizin Berlin, Berlin, Germany; ^2^Department of Physiology, University of Arizona, Tucson, AZ, United States; ^3^Deutsches Herzzentrum Berlin, Berlin, Germany

**Keywords:** blood flow, microcirculation, oxygen transport, structural adaptation, vascular remodeling

## Abstract

Diameters of microvessels undergo continuous structural adaptation in response to hemodynamic and metabolic stimuli. To ensure adequate flow distribution, metabolic responses are needed to increase diameters of vessels feeding poorly perfused regions. Possible modes of metabolic control include release of signaling substances from vessel walls, from the supplied tissue and from red blood cells (RBC). Here, a theoretical model was used to compare the abilities of these metabolic control modes to provide adequate tissue oxygenation, and to generate blood flow velocities in agreement with experimental observations. Structural adaptation of vessel diameters was simulated for an observed mesenteric network structure in the rat with 576 vessel segments. For each mode of metabolic control, resulting distributions of oxygen and deviations between simulated and experimentally observed flow velocities were analyzed. It was found that wall-derived and tissue-derived growth signals released in response to low oxygen levels could ensure adequate oxygen supply, but RBC-derived signals caused inefficient oxygenation. Closest agreement between predicted and observed flow velocities was obtained with wall-derived growth signals proportional to vessel length. Adaptation in response to oxygen-independent release of a metabolic signal substance from vessel walls or the supplied tissue was also shown to be effective for ensuring tissue oxygenation due to a dilution effect if growth signal substances are released into the blood. The present results suggest that metabolic signals responsible for structural adaptation of microvessel diameters are derived from vessel walls or from perivascular tissue.

## Introduction

**Figure 1:**
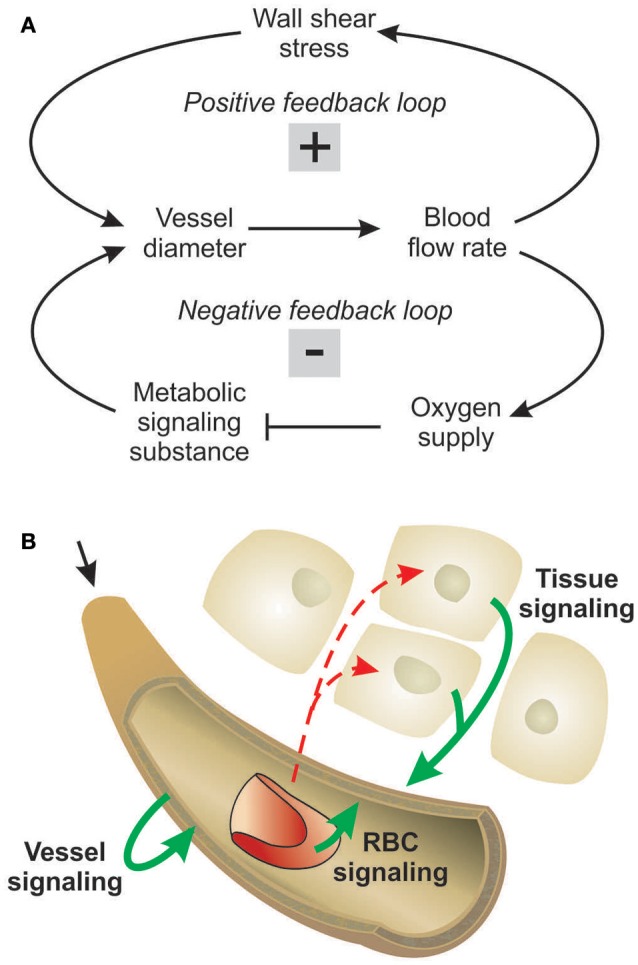
Concepts of metabolic signaling in vessel diameter adaptation. **(A)** Feedback loops in structural control of vessel diameters. Arrows and blunt ends indicate stimulatory and inhibitory actions respectively. Signs “+” and “–” in gray rectangles indicate positive and negative feedback regulation. Upper loop: an increase in vessel diameter causes an increase in blood flow and wall shear stress, which stimulates structural diameter increase. This positive feedback loop tends to destabilize vessel diameters. Lower loop: an increase in vessel diameter causes an increase in flow and improved tissue oxygenation. The resulting reduction in metabolic signal substance production evoking diameter increase causes structural diameter decrease. This negative feedback loop tends to stabilize vessel diameters. **(B)** Possible origins of metabolic signals. Local oxygen levels have been shown to evoke release of metabolic signal substances (solid arrows) from the vessel wall, from RBCs and/or from tissue cells. Dashed arrows indicate oxygen diffusion.

Structural adaptation (remodeling) of vessel luminal diameters is indispensable for generation and maintenance of functionally adequate microvascular networks. The structural diameter of a vessel is here defined as the internal diameter at a physiological transmural pressure in the absence of vascular tone. Under normal conditions, vessel diameters are determined by the structural diameter combined with any effects of smooth muscle cell tone. Several stimuli for structural diameter changes have been identified, including circumferential stress in the vascular wall, shear stress on the endothelial cell surface, local metabolic conditions and conducted responses propagated along vessel walls (Pries et al., 1998). Theoretical studies have shown that diameter adaptation in response to wall shear stress alone causes regression of complex network structures to single arterio-venous pathways (Rodbard, 1975; Hacking et al., 1996). This instability originates from a positive feedback mechanism: Vessels with higher blood flow experience higher wall shear stress, evoking a stimulus for diameter increase, which leads to further increase of blood flow (Figure 1A). Thus, a compensatory negative feedback mechanism is needed to maintain blood flow and to preserve parallel flow pathways. Such a negative feedback can be derived from local metabolic conditions, if low local blood flow or an imbalance between substrate supply and demand causes the generation of a signal for diameter increase (Figure 1A). Oxygen is a critical metabolite, and the metabolic signal is often assumed to be related to the local oxygen level. According to this concept, decreased oxygen partial pressures (PO_2_) induce increased release of metabolic signaling substance(s) which evokes structural increases of vessel diameters, increasing perfusion and restoring adequate PO_2_ values. Alternatively, decreased PO_2_ may induce decreased release of (vasoconstrictive) substances evoking diameter decrease.

Experimental studies of acute blood flow regulation have established the role of metabolic mechanisms in controlling diameters of arterioles and small arteries via changes of smooth muscle tone. These studies provide evidence for different sites of oxygen-dependent release of vasoactive substances, specifically the parenchymal tissue, the red blood cells (RBCs), and the vessel wall (Figure 1B). It has also been suggested that increasing PO_2_ may lead to increased release of vasoconstrictive mediators (Jackson, 1993; Lombard et al., 1999). As mediators of metabolic vasodilator signaling by the parenchymal tissue, substances including adenosine, CO_2_, ${\text{O}}_{2}^{-}$, phosphates, prostanoids and osmolality have been suggested (Berne, 1963; Broten et al., 1991; Golub and Pittman, 2013). RBCs were shown to deliver ATP from intracellular pools in response to the local saturation of hemoglobin with oxygen (Bergfeld and Forrester, 1992; Ellsworth et al., 1995). Additionally, nitric oxide (NO) as a metabolic signal substance is liberated from S-nitrosohemoglobin in proportion to the decline of hemoglobin saturation along a vessel (Jia et al., 1996; Stamler et al., 1997) or reduced from nitrite mediated by deoxyhemoglobin (Cosby et al., 2003; Nagababu et al., 2003). Possible metabolic signal substances released by the vessel wall or its immediately adjacent tissues include adenosine, NO, prostaglandins and endothelium-derived hyperpolarizing factor (Busse et al., 1983; Pohl and Busse, 1989; Messina et al., 1994; Earley et al., 2003). Several studies have shown that chronic vasoconstriction or vasodilation leads to corresponding structural changes in vessel diameter (Bakker et al., 2004; Jacobsen et al., 2008; Martinez-Lemus et al., 2009). Therefore, the various factors that have been proposed as contributing to acute flow regulation can also be considered as potential mediators of structural adaptation of vessel diameters.

While these studies have suggested potential mechanisms of metabolic signaling in structural adaptation, the relevance of these mechanisms and how they are integrated to achieve adequate diameter control remain to be determined. Therefore, the goal of the present study is to test several possible modes for metabolic structural control of vessel diameters, with respect to their ability to provide adequate tissue oxygenation and to generate blood flow velocities in agreement with experimental observations. A previously developed theoretical model (Reglin et al., 2009) is used to simulate vessel diameter adaptation in response to hemodynamic and metabolic stimuli for a rat mesentery microvascular network with 576 vessel segments where complete morphological and hemodynamic data sets were previously obtained from *in vivo* observations. By incorporating various assumed modes of metabolic control in the model, the effects of these control modes on network structure, flow and oxygenation were assessed. The tested modes include vessel-derived, tissue-derived and RBC-derived metabolic signals acting individually and in combination and oxygen-dependent and oxygen-independent generation of these signals.

## Materials and methods

### Experimental data

Details of the approach have been described previously (Pries, 1988). After approval by the University and State authorities for animal welfare, surgical and experimental procedures were carried out in accordance with the German Animal Protection Act. A microvascular network (total area ~25 mm^2^) of a male Wistar rat (~300 g body weight) in a fat-free and lymph vessel-free portion of the mesentery was recorded by intravital video microscopy in a scanning procedure lasting ~20 min. Upon finishing the experiment, the animal was sacrificed by an overdose of pentobarbital. The mesentery membrane is a relatively simple flat tissue, lacking some of the features of more complex parenchymal organs. However, it is reasonable to assume that a similar set of basic adaptation mechanisms is present in other tissues. Independent experiments with this preparation showed that arterioles exhibited no spontaneous smooth muscle tone. Thus, it can be assumed that the observed diameters reflect the structural diameters as defined above. To prevent development of tone during the scanning procedure, papaverine (10^−4^ mol/l) was continuously superfused.

Observation of the mesentery provided complete datasets including geometric and flow information for all vessel segments within the region under consideration. Diameter, length, hematocrit and blood flow velocity were measured off-line in all segments (vessel sections between branch points, *n* = 576) using a digital image analysis system (Pries et al., 2001). The network was supplied with blood by a main feeding arteriole (diameter 28 μm, blood flow 410 nl/min) and drained by a main venule (diameter 59 μm). Smaller secondary boundary vessels entered (*n* = 30) or left (*n* = 4) the observed region. The two-dimensional arrangement of vessels in the tissue was reconstructed and discretized by mapping the network onto a regular hexagonal (honeycomb) grid with edge length 40 μm, resulting in 4,740 vessel elements and 31,379 tissue elements (Reglin et al., 2009).

### Model approach

**Figure 2:**
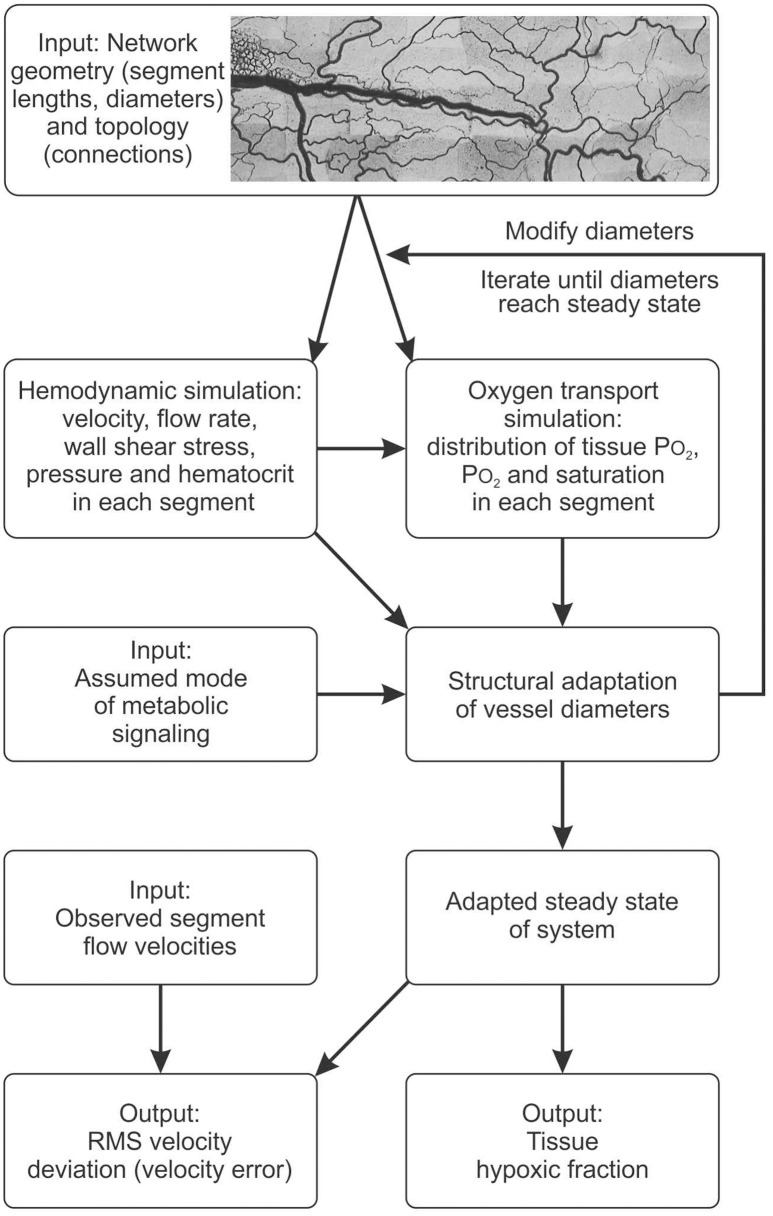
Schematic outline of modeling approach, indicating data input and assumptions used as input to the model, major steps in the computation, and resulting outputs. See text and supplement for details.

The experimental data on network structure and hemodynamics were used as the basis for theoretical simulations of vascular diameter adaptation of all vessels to hemodynamic and metabolic stimuli as described previously (Pries et al., 1998, 2001; Reglin et al., 2009) and as shown schematically in Figure 2. A more detailed description is given in the Supplementary Information. Physiological constants used in this model are given in Table 1. For each selected metabolic signaling mode, the resulting steady-state distributions of vessel diameters, hemodynamic parameters and oxygen levels are predicted using an iterative procedure. The observed vessel diameters are used as initial diameter estimates. Experimental data on flow conditions in boundary segments of the network are used to calculate flow rate, flow velocity, wall shear stress and discharge hematocrit for all segments (Pries et al., 1990). Hematocrits are calculated taking into account the effects of uneven red blood cell flux distribution at microvascular bifurcations with diverging flows (Pries et al., 1989). The oxygen distribution in the vessel network and the surrounding tissue is calculated from the geometry and arrangement of vessel and tissue elements, and from the blood flow and discharge hematocrit for each segment (Reglin et al., 2009). The oxygen level in the main feeding arteriole is assumed to be 95 mmHg. At each step in the iteration, vessel diameters are updated according an assumed set of adaptation rules using a model for structural vessel diameter adaptation (Pries et al., 2001). The adaptation rules, which are identical for all vessels of the network, prescribe vascular responses to adaptive stimuli. Increased wall shear stress, decreased transmural pressure and increased local concentration of a metabolic signal substance at the vessel wall are assumed to stimulate increase in vessel diameter. This procedure is repeated iteratively until a steady state is reached. No limitations are imposed with respect to maximal or minimal diameters achievable. Finally, the performance of each metabolic signaling mode is assessed in terms of the resulting oxygen supply to the tissue, i.e., the percentage of tissue area exhibiting a PO_2_ < 1 mmHg (tissue hypoxic fraction), and the deviation between experimentally measured and predicted flow velocities for all vessel segments, i.e., the velocity error *V*_*err*_ defined as the root mean square relative deviation of calculated from measured flow velocities. Velocity differences are a sensitive parameter since even small changes of diameters elicit large changes in flow velocity (Pries et al., 1998).

**Table 1:** Standard values of model parameters for oxygen transport and consumption.

**Description**	**Value**	**Unit**	**References**
**BLOOD PARAMETERS**			
Oxygen binding capacity of RBC	0.52	ml O_2_ ml^−1^	Clark et al., 1985
PO_2_ at half-maximal hemoglobin saturation	38	mmHg	Gray and Steadman, 1964
Hill equation exponent	3		Gray and Steadman, 1964
Solubility of oxygen in RBC	3.38 · 10^−5^	cm^3^ O_2_ cm^−3^ mmHg^−1^	Altman and Dittmer, 1971
Solubility of oxygen in plasma	3 · 10^−5^	cm^3^ O_2_ cm^−3^ mmHg^−1^	Hellums et al., 1996
Krogh diffusion coefficient in plasma	8.3 · 10^−10^	(cm^2^ s^−1^) (cm^3^ O_2_ cm^−3^ mmHg^−1^)	Hellums et al., 1996
**TISSUE PARAMETERS**			
Oxygen diffusion coefficient	1.04 · 10^−5^	cm^2^ s^−1^	Yaegashi et al., 1996
Solubility of oxygen	3 · 10^−5^	cm^3^ O_2_ cm^−3^ mmHg^−1^	Yaegashi et al., 1996
Krogh diffusion coefficient (K_t_ = D_t_ ·α_t_)	3.12 · 10^−10^	(cm^2^ s^−1^) (cm^3^ O_2_ cm^−3^ mmHg^−1^)	
Oxygen demand	0.01	cm^3^ O_2_ cm^−3^ min^−1^	Tsai et al., 1998; Golub et al., 2007
PO_2_ at half-maximal oxygen consumption	1	mmHg	Costa et al., 1997; Richmond et al., 1999
Thickness of the mesentery	20	μm	Barber et al., 1987

Three metabolic signaling modes were considered. A hypothetical signal substance was assumed to be generated in the vessel wall (vessel signaling), the parenchymal tissue (tissue signaling) and/or red blood cells (RBC signaling), at a rate that increases with decreasing oxygen level over a defined range of values. The production rate in the oxygen-dependent range is given in arbitrary units by:

(1) $$\begin{array}{r} J_{m}={\left[1-\left(\text{P}{\text{O}}_{2}\right)/\left({\text{PO}}_{2\text{ ref}}\right)\right]}^{\alpha }•G•\beta \;{\text{for PO}}_{2}\;\leq \;{\text{PO}}_{2\text{ ref}}\\ J_{m}=0\;for\;{\text{PO}}_{2}>{\text{PO}}_{2\text{ ref}} \end{array}$$

where PO_2 ref_ is a reference level of oxygen partial pressure. For RBC signaling, PO_2_ and PO_2 ref_ are replaced by So_2_ and So_2 ref_ in this equation. The parameter α defines the shape of the PO_2_ dependence of metabolic signal production. The standard value α = 1 gives linear dependence, and α > 1 (α < 1) gives increased PO_2_ sensitivity at low (high) PO_2_ values.

Reference PO_2_ values obtained previously by parameter optimization with the three signaling modes were used: 95 mmHg for vessel signaling, 115 mmHg for tissue signaling, and 92 mmHg, corresponding to SO_2 ref_ = 0.93, for RBC signaling (Reglin et al., 2009). The quantity *G* corresponds to the extent of the signal-producing structures. For tissue signaling, the volume of a tissue element is used. For RBC signaling, local tube hematocrit is used. For vessel signaling, several different measures of vessel extent were tested, as described below. The constant β is chosen for each mode to achieve the same total amount of metabolite released per time for all three modes, for the experimentally observed segment diameters.

The metabolic signal substance reaches the wall and lumen of vessel segments by diffusion in tissue signaling or is directly delivered into the flowing blood in vessel and RBC signaling. The metabolic signal substance entering the segment lumen locally in each vessel segment is added to the amount of metabolic signal substance convected from upstream regions, assuming no decay. In addition to downstream convection, the metabolic signal information is assumed to be propagated upstream along vessel walls by vascular conducted responses (Pries et al., 1998, 2001).

### Variation of assumed metabolic signaling mode

For all assumed metabolic signaling modes, adjustable model parameters were optimized to minimize the velocity error *V*_*err*_. For each parameter setting, the tissue hypoxic fraction was determined.

The vessel signaling mode served as the reference for additional analysis, since this mode was previously found to result in lower velocity errors than tissue and RBC signaling modes (Reglin et al., 2009). For this mode, several alternatives for the quantity *G* in Equation (1) were tested, as follows: (i) no dependence on geometry or flow (*G* = 1); (ii) vessel length (*G* = *L*, standard case); (iii) vessel surface area (*G* = *L*·*D*); (iv) vessel volume (*G* = *L*·*D*^2^); (v) vessel blood flow (*G* = *D*^2^·*v*); where *L* = vessel length, *D* = vessel diameter, *v* = blood flow velocity.

To test the effect of the assumed dependence of metabolic signal production on oxygen level, two types of dependence were considered in vessel signaling. (i) Production rate is fixed for PO_2_ values below a threshold and decreases linearly with increasing PO_2_ above the threshold according to Equation (1). (ii) Production rate decreases with increasing PO_2_ below a threshold and is fixed for PO_2_ values above the threshold. In each case, the threshold PO_2 ref_ was varied from 0 to 95 mmHg.

As already mentioned, multiple mechanisms for acute metabolic flow regulation have been identified, and these may contribute to structural adaptation of vessel diameters. To test the effect of combining different modes of metabolic signaling, the three modes were combined linearly with weighting factors adding up to 1, so as to maintain the overall strength of metabolic signaling. Individual weighting factors were systematically varied in steps of 0.1. The rates of production of metabolic signal substances by the different modes were determined and the concentration of signal substance in a given vessel segment was multiplied with the respective weighting factor. The summed local concentration was then used to calculate the metabolic stimulus. Simulations were performed assuming oxygen dependent production of metabolic signaling (according to Equation 1) and also with oxygen-independent production (according to *J*_*m*_ = *G* β).

## Results

### Effects of combining vessel, tissue, and RBC signaling modes

**Figure 3:**
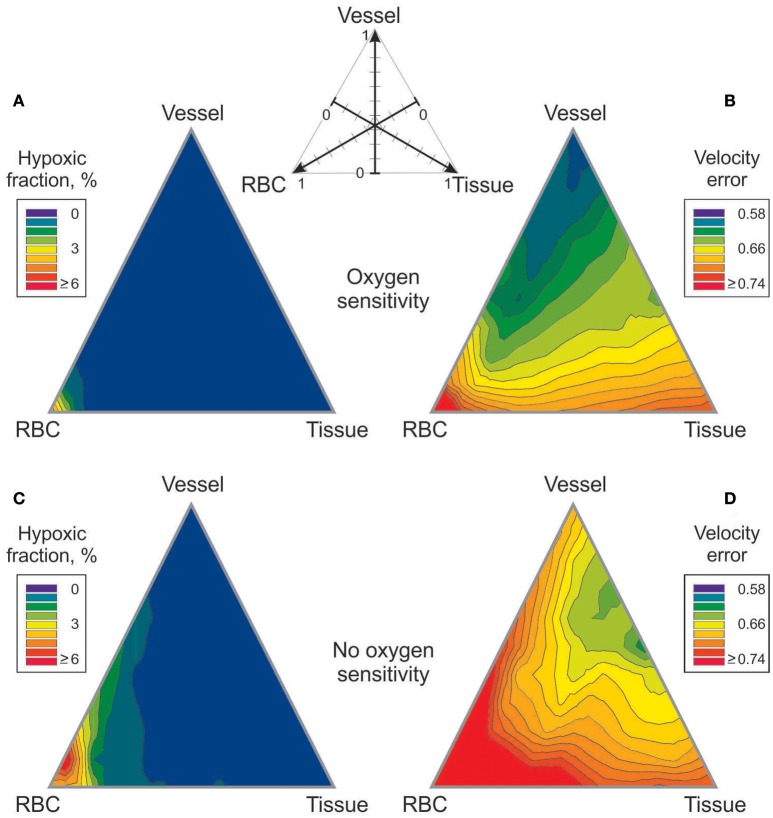
Effect of combining metabolic signaling by vessel signaling, tissue signaling and RBC signaling modes on tissue hypoxic fraction **(A,C)** and on velocity error **(B,D)**. The three modes were weighted according to the scheme shown at top, with the weights adding up to 1. **(A,B)** Results with oxygen dependent generation of metabolic signal substance. **(C,D)** Results with oxygen independent generation of metabolic signal substance.

The effects of metabolic signaling by combinations of vessel, tissue and RBC modes on hypoxic fraction and velocity error are shown in Figure 3. With either oxygen-sensitive or oxygen-insensitive signaling, hypoxic fractions below 1% are achieved for all combinations except those dominated by RBC signaling (Figures 3A,C). Thus, any combination with a significant component of vessel and/or tissue signaling yields vessel diameter and blood flow distributions resulting in low tissue hypoxia levels. Combinations dominated by RBC signaling also yield high levels of velocity error, *V*_*err*_ (Figure 3B). The lowest values of velocity error, indicating closest agreement with experimental results, are obtained with oxygen-sensitive vessel signaling (*V*_*err*_ = 0.58). When oxygen-insensitive signaling is assumed, a combination of vessel and tissue signaling gives the lowest *V*_*err*_ (0.61) (Figure 3D). These results suggest that the metabolic control of the experimentally observed vascular structure was governed by an oxygen-sensitive vessel signaling mode. No other combination of signaling modes gave a lower *V*_*err*_. In further simulations, oxygen-sensitive vessel signaling and RBC signaling were combined with oxygen-insensitive tissue signaling. The results (Figure S1) differed only slightly for those with oxygen-sensitive tissue signaling.

### Effects of signal-producing structure (vessel signaling)

**Figure 4:**
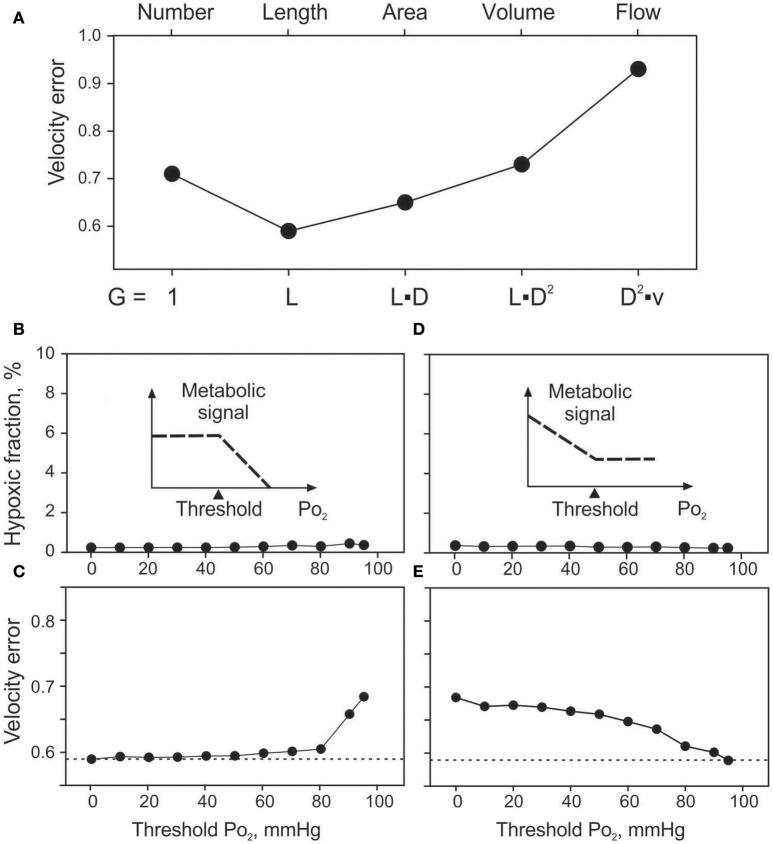
Effects of assumed vessel signaling mechanisms on tissue oxygenation and velocity error. **(A)**. Effect of the extent of signal producing structures on velocity error (*V*_*err*_). Changing factor *G* in equation 1, sensitivity to vessel number (*G* = 1), length, surface area, volume and flow is modeled. **(B,C)**. Effects of oxygen sensitivity range in vessel signaling, with sensitivity only above the threshold level up to 95 mmHg (see inset). Effects of threshold PO_2_ on hypoxic fraction **(B)** and *V*_*err*_ **(C). (D,E)**. Effects of oxygen sensitivity range in vessel signaling, with sensitivity only below the threshold PO_2_ level down to 0 mmHg (see insets). Effects of threshold PO_2_ on hypoxic fraction **(D)** and *V*_*err*_ **(E)**.

Effects of the assumed geometrical dependence of metabolic signal production (*G* in Equation 1) on velocity error (*V*_*err*_) for vessel signaling are summarized in Figure 4A. Assuming no dependence of production on geometry or flow (*G* = 1) in a given vessel segment resulted in *V*_*err*_ = 0.71. The minimal velocity error (0.59) was obtained assuming production proportional to vessel length. Signal production proportional to vessel surface area, vessel volume or vessel blood flow gave successively higher values of *V*_*err*_. Tissue hypoxic fraction (data not shown) exhibited a similar behavior with values of 0.54, 0.24, 0.30, 1.19, and 8.95% for the five cases. Length dependence was chosen as the standard for vessel signaling for all other simulations.

### Effects of restricting range of oxygen sensitivity

**Figure 5:**
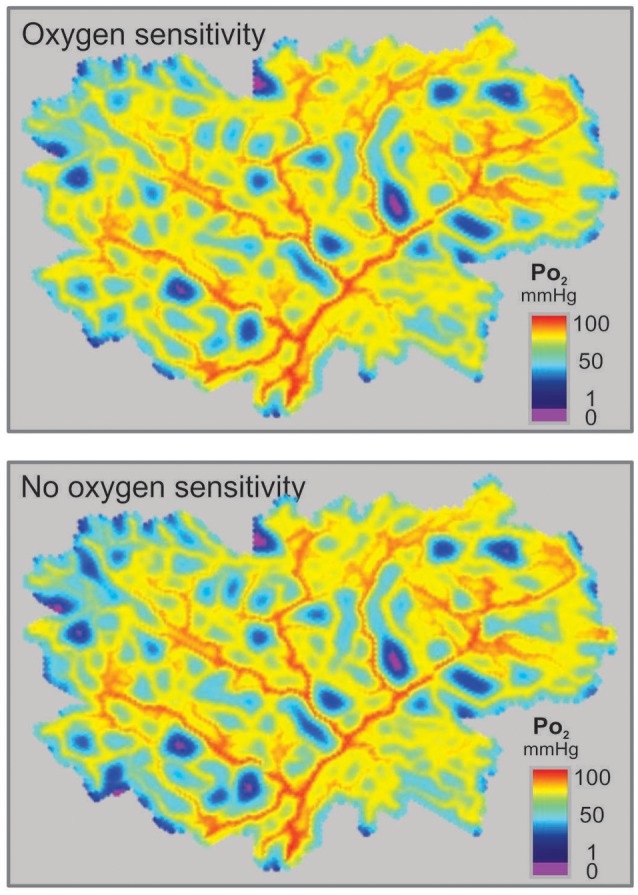
Distributions of simulated PO_2_ in a mesenteric section supplied by a microvascular network with vessel diameters resulting from simulated adaptation assuming oxygen sensitivity of vascular adaptation **(Top)** or no oxygen sensitivity **(Bottom)**.

The minimal values of *V*_*err*_ and hypoxic fraction were obtained when the metabolic signal was assumed to be sensitive to PO_2_ over the entire range from 0 to 95 mmHg. This corresponds to thresholds of 0 mmHg and 95 mmHg respectively for the two types of oxygen sensitivity considered. However, in each case, altering the threshold for oxygen sensitivity resulted in only slight increases in hypoxic fraction (Figures 4B,D), even when oxygen sensitivity was eliminated. The computed oxygen distributions in the tissue with and without oxygen sensitivity are shown in Figure 5. This unexpected finding that oxygen sensitivity was not required for effective tissue oxygenation is discussed below. The velocity error remained low as long as oxygen sensitivity was retained for PO_2_ levels above 80 mmHg (*V*_*err*_ 0.61 for a threshold PO_2_ of 80 mmHg), but increased when oxygen sensitivity above 80 mmHg was removed from the model (Figures 4C,E).

Further simulations were performed with combinations of two signaling modes, where one mode was assumed to exhibit PO_2_ sensitivity in a high PO_2_ range and the other in a low PO_2_ range. The results (Figure S2) showed that low velocity errors are achieved only when wall signaling is included, with oxygen sensitivity in the upper range of PO_2_ values. Additional simulations were performed (data not shown), in which oxygen sensitivity was assumed to depend on intravascular pressure, wall shear stress or vessel diameter, or was restricted to arterioles. Also, the exponent α in Equation (1) was varied from 1 to between 0.5 and 3. All approaches led to higher *V*_*err*_ levels compared to standard conditions (α = 1).

## Discussion

The goal of the present work is to gain insight into the mechanisms governing structural responses of microvessel diameters to metabolic signals. The approach depends on the assumption that fully adapted inner vessel diameters represent the outcome of biological mechanisms that control structural adaptation in response to metabolic and hemodynamic signals, so as to provide a biologically adequate distribution of blood flow in the vascular network. Therefore, simulating possible mechanisms and testing their ability to generate results consistent with experimental observations allows testing of hypotheses regarding these mechanisms of adaptation. The simulations of diameter adaptation are based on microvascular network data derived from the mesentery, a tissue that does not experience large changes in metabolic demand over time, and from adult animals. Therefore, the observed vessel morphology can reasonably be assumed to represent a fully adapted steady state of the system. The experimental vasculature studied here did not exhibit tone during the experiment (Pries et al., 1998), and so vessel diameters represent solely the result of structural adaptation. In the presence of tone, arteriolar diameters would reflect the combined effect of structural (maximally dilated) diameter and superimposed smooth muscle contraction.

In this study, we assessed different assumed mechanisms of structural adaptation in terms of two parameters: predicted hypoxic fraction; and overall deviation between predicted and observed velocities (*V*_*err*_). The hypoxic fraction is considered as a measure of the potential of the assumed mechanism to meet the functional needs of the tissue for oxygen supply, while *V*_*err*_ indicates deviation from experimentally observed conditions. The errors in the experimental measurements of vessel diameters, flow velocities, etc., and the simplifying assumptions of the model result in a basic component of *V*_*err*_ which is relatively large compared to the variations in *V*_*err*_ found with different assumed mechanisms (Pries et al., 1994). However, the remaining component of *V*_*err*_ is sensitive to changes in the assumed adaptive mechanisms, and minimization of *V*_*err*_ may indicate which mechanisms are likely present in the physiological system.

In previous work (Reglin et al., 2009), we considered the same three modes of metabolic control of vascular diameters, namely vessel signaling, tissue signaling and RBC signaling, each acting individually, and showed that vessel signaling led to minimal tissue hypoxia and closest agreement between predicted and observed vessel flow velocities in individual segments (minimal *V*_*err*_). Those results were confirmed in the present study, where the velocity error was lowest with vessel signaling mode, higher with tissue signaling mode, and highest with RBC signaling mode (Figure 3). The previous study did not consider the possibility that a combination of signaling modes could lead to lower levels of tissue hypoxia and *V*_*err*_ than could be achieved by any one mechanism acting alone. The present results show that this is not the case when oxygen-sensitive signaling is considered (Figure 3B). In the present study, we also considered cases in which all three signaling modes are insensitive to oxygen levels. The results for hypoxic fraction are similar to those with oxygen-sensitive signaling, in that hypoxic fraction was low with vessel or tissue signaling but high with RBC signaling (Figure 3C). In these case, *V*_*err*_ levels were somewhat higher and the minimum was found for a combination of tissue and vessel signaling (Figure 3D).

In the present study, we explored the dependence of vessel signaling on the assumed signal-producing structure, by considering signals generated in proportion to the number, length, surface area, volume, or flow rate in each vessel. While it might be expected that signals are generated in proportion to vessel surface area (i.e., endothelial cell area), the best match between model predictions and experimental data was obtained for vessel signaling in proportion to vessel length (Figure 4A). Signal generation in proportion to surface area has the consequence that the signal decreases with decreasing vessel diameter, which diminishes the ability of low-flow vessels to restore adequate flow. This may account for the advantageous behavior of length-dependent signaling. One possible interpretation of this result is that the signal is generated from a tissue sleeve of constant outer radius adjacent to the vessel, such that the volume of signal-generating tissue is proportional to the vessel length. Such behavior could result from mechanisms limiting the effective diffusion distance of signaling molecules such as adenosine (Deussen, 2000), VEGF (Ji et al., 2007) or HETE (Lombard et al., 1999). This “tissue sleeve” hypothesis might explain experimental observations where PO_2_ sensitivity was lost after complete dissection of perivascular tissue from arterioles *in situ* (Jackson, 1987) while being preserved if even small amounts of perivascular tissue remained in contact with the vessel (Jackson and Duling, 1983).

**Figure 6:**
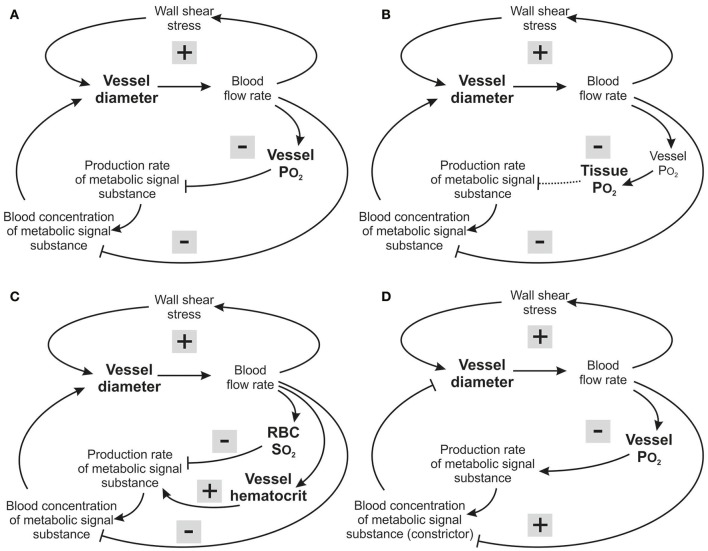
Feedback loops involved in structural control of structural vessel diameters, under different assumed metabolic signaling modes. Arrows and blunt ends indicate stimulatory and inhibitory action. Signs “+” and “–” in gray rectangles indicate positive and negative feedback regulation. Upper loop: hemodynamic feedback. Lower loops: metabolic feedback. In all cases, the growth response to wall shear stress produces a positive hemodynamic feedback loop, which must be counteracted by negative feedback from metabolic responses to maintain stability. **(A)** Vessel signaling. Increased blood flow causes increased intravascular PO_2_ which reduces production of the metabolic signaling substance, resulting in a negative feedback loop (upper metabolic loop). In addition, the dilution effect (lower metabolic loop) introduces a negative feedback that operates independently of oxygen sensitivity. **(B)** Tissue signaling. Increased blood flow causes increased intravascular PO_2_, and hence increased tissue PO_2_, which reduces production of the metabolic signaling substance, resulting in a negative feedback loop which, however will be compromised for situations where the maximal oxygen diffusion distance is exceeded (dashed arrow). The dilution effect is again present. **(C)** RBC signaling. Increased blood flow causes increased RBC hemoglobin oxygen saturation, reducing production of the metabolic signaling substance, resulting in a negative feedback loop (upper metabolic loop). However, decreasing vessel diameter and blood flow decrease the local concentration of RBCs due to unequal RBC distribution at microvascular bifurcations, and this introduces a destabilizing positive feedback effect (middle metabolic loop). The dilution effect is again present (lower metabolic loop). **(D)** Vessel signaling with a *vasoconstrictor* signaling substance. Increased blood flow causes increased intravascular PO_2_, which increases production of the metabolic vasoconstrictor signaling substance, resulting in a negative feedback loop. However, increased blood flow also reduces the concentration of the metabolic signaling substance in the blood by the dilution effect, which introduces a destabilizing positive feedback.

The role of oxygen sensitivity in metabolic signaling was investigated by restricting the range of PO_2_ values for which metabolic signals are produced in an oxygen-dependent fashion. Sensitivity in the high PO_2_ range was shown to have the greatest impact in reducing *V*_*err*_ (Figures 5D,E) while varying oxygen sensitivity at lower PO_2_ levels had little effect (Figures 5B,C). Even in the complete absence of oxygen sensitivity, relatively low velocity errors were obtained with vessel signaling. Thus, the results of the present study surprisingly suggest that functionally adequate vessel diameter distributions can be achieved with restricted or absent oxygen sensitivity. This finding can be understood in terms of feedback mechanisms involved in control of vessel diameters (Figure 6A). Independent of oxygen sensitivity, negative feedback is provided by the “dilution effect” (Reglin and Pries, 2014) for signal substances released into the blood that evoke vessel diameter increase: The concentration in the blood of a substance that diffuses into the vessel at a given rate increases inversely with decreasing volume flow rate. In low flow vessels, the higher substance concentration may thus evoke a stronger signal for diameter increase, providing a simple and robust mechanism for adaptation to maintain adequate flow in all vessels, provided that the existing network structure provides adequate vessel density. This mechanism may operate in tissues such as the lung that are not dependent on blood for oxygen supply. However, mechanisms involving sensitivity to oxygen must play a key role in enabling adaptation of vascular networks to short and long term changes in the oxygen supply-to-demand ratio of the tissue, including stimulation of angiogenesis to increase vascular density when oxygen demand increases (Secomb et al., 2013; Reglin and Pries, 2014).

Insight into the possible reasons for this finding can be obtained by considering the feedback loops involved in each signaling mode (Figure 6). In vessel signaling, the dilution effect and PO_2_-dependent signaling act together to provide robust negative feedback regulation, with vessel diameter increasing in response to low flow and low PO_2_ (Figure 6A). This provides a likely explanation for the effectiveness of vessel signaling in producing low levels of tissue hypoxia and velocity error.

In tissue signaling, the situation is similar to that in vessel signaling, with both the dilution effect and PO_2_-based signaling providing negative feedback regulation (Figure 6B) but somewhat higher velocity error (Figure 3B). This may be due to partial decoupling of the feedback, as oxygenation of tissue cells is determined both by vascular perfusion and diffusion distance. If vessel density is too low, increase of vascular diameter caused by metabolic signals released by the tissue cannot lead to adequate tissue oxygenation if the distance from microvessels to cells within the tissue exceeds the maximal oxygen diffusion distance (depending on tissue oxygen consumption). In this situation, inadequately supplied tissue cells would continue to produce increased levels of the metabolic signal substance despite the diameter increase of existing vessels, compromising the metabolic negative feedback signaling (Reglin et al., 2009).

In RBC signaling, both the dilution effect and the SO_2_-based signaling provide negative feedback regulation (Figure 6C). However, in this case the metabolic substances are generated in proportion to local tube hematocrit (*H*_*T*_). As a consequence of the unequal partition of hematocrit in diverging microvascular bifurcations, vessels with lower flow also tend to receive lower hematocrit (Pries et al., 1989). This effect reduces the amount of RBC-derived signal substance in a low-flow vessel, creating a destabilizing positive feedback loop (Reglin et al., 2009; Figure 6C). Therefore, RBC signaling provides a less effective mechanism for ensuring tissue oxygenation, relative to vessel and tissue signaling. An analogous conclusion was reached in the context of acute flow regulation (Roy et al., 2012).

Low PO_2_ was considered to lead to an increased release of dilator substances in the models discussed so far. Alternatively, decreasing PO_2_ could lead to a declining release of vasoconstrictive mediators, as has been shown for release of leukotrienes or 20-HETE *in vivo* (Jackson, 1993; Lombard et al., 1999). In this case, shown schematically in Figure 6D, an increase in flow results in a reduced concentration of the vasoconstrictor by the dilution effect, creating a positive feedback mechanism that leads to further flow increase and works against uniform tissue oxygenation. Therefore, it is unlikely that vasoconstrictive mediators can play a primary role in structural adaptation of diameters in response to metabolic stimuli.

When comparing different signaling modes, the constants β that specify the signal strength must be chosen. Here, the values were chosen to give the same total metabolite release rate for all three modes. The possibility arises that other assumptions might lead to different conclusions. For instance a higher value of β might give improved tissue oxygenation with RBC signaling. In all cases considered, however, RBC signaling gave relatively high levels of hypoxia and *V*_*err*_. It was not possible to reduce *V*_*err*_ to levels comparable to those found with the vessel or tissue mode by varying the metabolic feedback strength for RBC signaling Reglin et al., 2009. This is an expected consequence of the positive feedback mechanism shown in Figure 6C, which would only be strengthened by increasing β.

In summary, theoretical models have been developed for structural control of microvessel diameters by metabolic signals, and the predictions were compared with *in vivo* experimental observations. With this approach, a variety of hypotheses regarding the underlying mechanisms have been tested. The results indicate that growth signals generated in the vessel wall or an adjacent tissue sleeve in response to reduced PO_2_ are likely to play a dominant role in the metabolic control of vessel diameters. In this context, oxygen sensitivity in the high PO_2_ ranges seems to be especially relevant to achieve realistic diameter distributions. Growth signals derived from the entire tissue may also play a role but give results that agree less well with experimental observations. On the other hand, such signals presumably play an important role in stimulating angiogenesis when vascular density is inadequate. In contrast, RBC signaling results in inadequate oxygen distribution, with significant fraction (~5%) of hypoxic tissue. With decreasing flow, vessel hematocrit decreases as a consequence of unequal hematocrit partition in diverging bifurcations. The reduction of the concentration of RBCs releasing metabolic signal substances creates a positive feedback loop. Also, signaling based on a vasoconstrictor released at high oxygen levels is unlikely to be effective as it likewise creates a positive feedback loop. The ability of blood-borne growth signals generated in response to reduced oxygen levels to regulate vessel diameters is enhanced by the dilution effect, which causes a reduction of concentration with increasing flow, providing a negative feedback mechanism. This mechanism is effective even if the generation of the metabolic signal substance is independent of oxygen levels.

These results may suggest new targets for experimental studies of metabolic control of vessel luminal diameter. Candidate molecules for metabolic control of diameters are likely to be produced by or in the close vicinity of the vessel wall. While it is likely that the generation of these substances is PO_2_-dependent, growth signals whose generation has no oxygen sensitivity or a limited range of sensitivity may contribute to diameter control, as a consequence of the dilution effect.

## Author contributions

BR, TS, and AP conceived main ideas of the study; BR conducted modeling and drafted the manuscript; BR, TS, and AP analyzed and interpreted the data, and prepared the final manuscript.

### Conflict of interest statement

The authors declare that the research was conducted in the absence of any commercial or financial relationships that could be construed as a potential conflict of interest.
