# Supplementary material for: Structural Control of Microvessel Diameters: Origins of Metabolic Signals
Source: Front Physiol. 2017 Oct 24;8:813. doi: 10.3389/fphys.2017.00813 (PMC5660852; doi:10.3389/fphys.2017.00813)
Supplement: Supplementary file 1 [file DataSheet1.PDF]

Supplementary Material

Structural control of microvessel diameters:

Origins of metabolic signals

Bettina Reglin, Timothy W. Secomb and *Axel R. Pries

***Correspondence:** Axel.Pries@charite.de

Material and Methods: Details of Modeling Approach

Network hemodynamics

Input data for the calculation of vascular network hemodynamics comprise morphological data (diameter and length) for each vessel segment and data on network topology (segment connection matrix) as well as boundary conditions for all segments entering (flow or pressure, discharge hematocrit, oxygen saturation) or leaving (flow or pressure) the network. Details of the hemodynamic model have been described earlier (1). The flow resistance of each vessel segment is estimated, using the experimentally determined variation of apparent viscosity of blood with vessel diameter and hematocrit (Fahraeus-Lindqvist effect) (1), and taking into account the phase separation of red blood cells and plasma in diverging bifurcations (2). Segment resistances are then used to compute the distribution of blood flow and pressures in the whole network. The updated flow distribution leads to a change in phase separation and thus segment hematocrit and viscosity, which in turn is used in the next computation of segment blood flow and pressure distribution. Both steps are repeated until stable values are achieved. Values of wall shear stress in each segment are then calculated from the pressure, diameter and length values.

Oxygen distribution

Details of the oxygen transport model, which is based on a finite difference approach, have been described before (3). The oxygen distribution in the vessel network and the surrounding tissue is calculated from the morphological and topological data, and from the convective transport in the network (blood flow and discharge hematocrit for each segment). The convective oxygen flux of each vessel is determined considering oxygen bound to haemoglobin and dissolved in RBCs and plasma. Haemoglobin oxygen saturation is calculated according to the Hill equation.

For simulation of oxygen diffusion, all vessel segments of the planar microvascular network were mapped onto a regular hexagonal “honeycomb” grid with a hexagonal edge length of 40 µm. Each hexagon was divided into six equilateral triangles defining tissue elements. This discretization of the mesenteric network resulted in 4,740 vessel elements and 31,379 tissue elements. Oxygen diffusion is simulated between these elements according to Fick's law of diffusion as a bidirectional exchange process between the tissue and all types of vessels (arterioles, capillaries, venules), and within the

tissue. Within the tissue, oxygen consumption, diffusivity and solubility are assumed spatially uniform. The oxygen consumption rate of the tissue is assumed to depend on PO_2 according to Michaelis-Menten kinetics.

Oxygen distribution in the vascular network and the surrounding tissue is assessed by iteratively calculating oxygen convection in the vascular network, oxygen diffusion from the vessels into the tissue and within the tissue and oxygen consumption by the tissue, until stable values are achieved. The model obeys mass conservation, and the difference between total oxygen inflow and outflow equals the summed oxygen consumption by the tissue assuming zero diffusive flux at the outer boundaries of the tissue region considered.

Adaptation model

The adaptation model (4, 5) is based on the hypothesis that the structural diameter of each vessel segment is determined by responses to stimuli derived from local hemodynamic and metabolic conditions or signals. Diameter changes triggered by these stimuli are assumed to be governed by the same set of rules in all segments of the network. The diameter adaptation process is inherently unbounded, and the final (steady state) diameter of a given vessel is only determined by the adaptive stimuli, derived from the surrounding conditions, and the assumed adaptation rules.

For secondary boundaries, values for oxygen saturation and flux of metabolic signal substance (inputs), and conducted signals (outputs) were assigned. To minimize possible bias due to these assigned values on simulation results, the respective values were estimated to correspond to values for main feeding and draining segments.

For all simulations, the following boundary conditions were set to correspond to *in vivo* conditions: oxygen partial pressure in the main feeding arteriole (95 mmHg, estimated); discharge hematocrit in input boundary segments (individually measured for each segment); transmural pressure in the main draining venule (13.8 mmHg, according to measurements (1)); flow rates in all other segments entering or leaving the network (calculated from measured flow velocities and vessel diameters).

In each adaptation step, the diameters of all vessel segments were updated in parallel as described below. Each diameter change leads to a change in the distribution of flow resistances, necessitating recalculation of network hemodynamics and oxygen distribution. The results of this calculation, in turn, define the conditions for the next diameter adaptation step. Vessel diameter adaptation is thus simulated as an iterative process, alternately updating hemodynamic and metabolic variables and vessel diameters. The step size is controlled by the time step Δt. After reaching convergence, the net adaptive stimulus S_{tot} (eq. S1) approaches zero for each vessel segment. In the present study, we did not investigate dynamic changes and thus the time step duration is an arbitrary parameter. This iteration was repeated until convergence was achieved (segment average diameter change $< 10^{-5}$ µm).

For each vessel segment, the hemodynamic and metabolic stimuli are summed to obtain the net adaptive stimulus, S_{tot}, which determines the relative change of the vessel diameter per time step (Δt):

$$\Delta D/D = S_{tot} \cdot \Delta t \tag{S1}$$

and

$$S_{tot} = k_h\,(S_\tau + k_p S_p) + k_m\,(S_m + k_c S_c) - k_s. \qquad \text{(S2)}$$

where the hemodynamic stimuli (S_τ, S_p) are derived from local wall shear stress (τ_w) and transmural pressure (P), respectively. The metabolic stimulus (S_m) is derived from the local concentration of a metabolic signal substance at the vessel wall, which also contributes to a conducted stimulus (S_c, see below). The constant k_h determines the impact of hemodynamic stimuli for diameter adjustment, and k_p is used to weigh the relative impact of pressure to shear stress. Because the net adaptive stimulus S_{tot} is zero in the equilibrium state, only the relative strength of hemodynamic and metabolic stimuli is relevant, and k_h was set to 1. The relative impact of metabolic stimuli compared to hemodynamic stimuli is thus determined by k_m while k_c controls the relation of conducted stimuli to stimuli derived from local metabolite concentration. The parameter k_s represents a shrinking tendency of vessels in the absence of positive growth stimuli.

S_τ is calculated from wall shear stress, τ_w, as log(τ_w), i.e. increased wall shear stress stimulates diameter increase. S_p is derived from transmural pressure, P, as $S_p = -\log\,[\tau_e(p)\,/\,\varepsilon]$ where $\tau_e(\mathrm{p}) = [100 - 86\,\exp(-5{,}000\,\{\log\,[\log\,(p\,/\,\varepsilon)]\}^{5.4})]$ describes the shear stress set point as a function of P according to experimental data (6). The dimensional constants ε (= 1 mmHg here) and in the following equations are introduced to ensure correct dimensions. Thus, increased transmural pressure evokes diameter decrease.

A metabolic signaling substance is assumed to cause vessel diameter increase. According to experimental studies on vascular responses to local oxygenation, three metabolic signaling modes were considered, with production of the signaling substance in red blood cells (RBC signaling), the vessel wall (vessel signaling) and the parenchymal tissue (tissue signaling).

Rules for release of the metabolic signaling substance exhibit the same general structure. The signal substance is assumed to be released at a rate J_m (arbitrary units) according to eq. S3 (corresponding to eq. 1 in the main text). Release rate depends on:

1. Local P_{O_2} (S_{O_2} for RBC signaling). The oxygen dependent hypothetical vasoactive metabolic signal substance is generated if P_{O_2} (S_{O_2}) in the respective oxygen sensing structure falls below a reference level ($P_{O_2\,ref}$, $S_{O_2\,ref}$). Below this level, an increase of production rate with decreasing oxygen content is assumed, with α defining the shape of the P_{O_2} dependency of metabolic signal production: $\alpha > 1$ ($\alpha < 1$) increases P_{O_2} sensitivity at low (high) P_{O_2} ranges relative to a linear relation ($\alpha = 1$, used as standard). The oxygen dependent signal is set to zero if the present value exceeds the reference value.

2. The extent of the substance releasing structure, G.

3. A scaling constant β is introduced to guarantee that under baseline conditions, i.e. assuming experimental segment diameters, the total amount of metabolite released per time is the same for all tested modes.

$$J_m = \left(1 - \frac{P_{O_2}}{P_{O_2\,ref}}\right)^{\alpha} \cdot G \cdot \beta \qquad \text{for } P_{O_2} \le P_{O_2\,ref}$$

$$J_m = 0 \qquad \text{for } P_{O_2} > P_{O_2\,ref.} \qquad \text{(S3)}$$

In the standard tissue signaling mode, the signal substance is released from each tissue element according to

$$J_m = \left(1 - \frac{P_{O_2\,tis}}{P_{O_2\,ref}}\right)^a \bullet V_{tis} \bullet \beta \tag{S4}$$

where the volume of the substance releasing structure corresponds to the volume of the tissue element (V_{tis}). Thus, parallel with P_{O_2} changes from the vessel wall into the tissue, the model will result in tissue inhomogeneity in metabolic signal release.

In the standard vessel signaling mode, substance release was assumed to occur in proportion to segment length (L) with substance flux added to the blood calculated as

$$J_m = \left(1 - \frac{P_{O_2\,seg}}{P_{O_2\,ref}}\right)^a \bullet L \bullet \beta. \tag{S5}$$

The standard RBC signaling mode corresponds to experimental findings on ATP release from erythrocytes (7). The signal substance is released in response to oxygen saturation and in proportion to local tube hematocrit (H_T) according to

$$J_m = \left(1 - \frac{S_{O_2\,seg}}{S_{O_2\,ref}}\right)^a \bullet V_{seg} \bullet H_T \bullet \beta \tag{S6}$$

where $S_{O_2\,seg} / S_{O_2\,ref}$ is the segmental oxygen saturation (for the average P_{O_2} of the segment) relative to oxygen saturation for the reference oxygen partial pressure, and V_{seg} is the intravascular volume.

The metabolic signal substance reaches the wall and lumen of vessel segments by diffusion (tissue signaling) or is directly delivered into the flowing blood (vessel signaling, RBC signaling). The metabolic signal substance locally entering each vessel segment ($J_{m,seg}$) is added to the amount of metabolic signal substance convected from upstream regions ($J_m{}^{up}{}_{seg}$) resulting in substance flux at the downstream end of the segment ($J_m{}^{down}{}_{seg}$) according to

$$J_m{}^{down}{}_{seg} = J_m{}^{up}{}_{seg} + J_{m,seg}. \tag{S7}$$

At vascular branch points, the metabolic signal substance is distributed in proportion to blood flow. No decay of the signal substance was assumed. The local concentration of metabolic signal substance in the blood (c_m) is calculated from the amount of signal substance transported by intravascular convection as

$$c_{m,seg} = \frac{J_{m,\,seg}}{Q + Q_{ref}} \tag{S8}$$

where Q is segment blood flow. Q_{ref} is a small constant included to keep concentration values bounded for very low segment blood flow. For tissue signaling and RBC signaling modes $c_m = c_{m,seg}$ while for tissue signaling it is given as $c_m = (c_{m,seg} + c_{m,tis}) / 2$.

The metabolic stimulus (S_m) is calculated from the local concentration of metabolic signal substance in the blood (c_m) according to

$$S_m = \log\left[1 + \frac{c_m}{\varepsilon}\right]. \qquad \text{(S9)}$$

In line with experimental and theoretical findings (8, 9), a conduction of metabolic information upstream along vessel walls throughout the entire network is assumed (10): A local conducted signal (J_c, dimensionless) is generated in each vessel in proportion to the local metabolic stimulus and to segment length according to

$$J_c = S_m\, L\, \varepsilon \qquad \text{(S10)}$$

J_c is added to the conducted signal reaching the segment from downstream parts of the vessel network (J_c^{down}) leading to a conducted signal on the upstream end of the segment (J_c^{up}) according to

$$J_c^{up} = J_c^{down} + J_c\,. \qquad \text{(S11)}$$

At converging bifurcations the signal is equally distributed to the (upstream) feeding segments independent of their blood flow, in diverging bifurcations signals of the (downstream) daughter segments are summed. The conducted stimulus (S_c) for diameter adaptation based on the mid-segment conducted signal is defined as

$$S_c = \frac{J_c}{J_c + J_{ref}} \qquad \text{(S12)}$$

where J_{ref} is a reference conducted signal resulting from parameter optimization.

1.1 Supplementary Figures

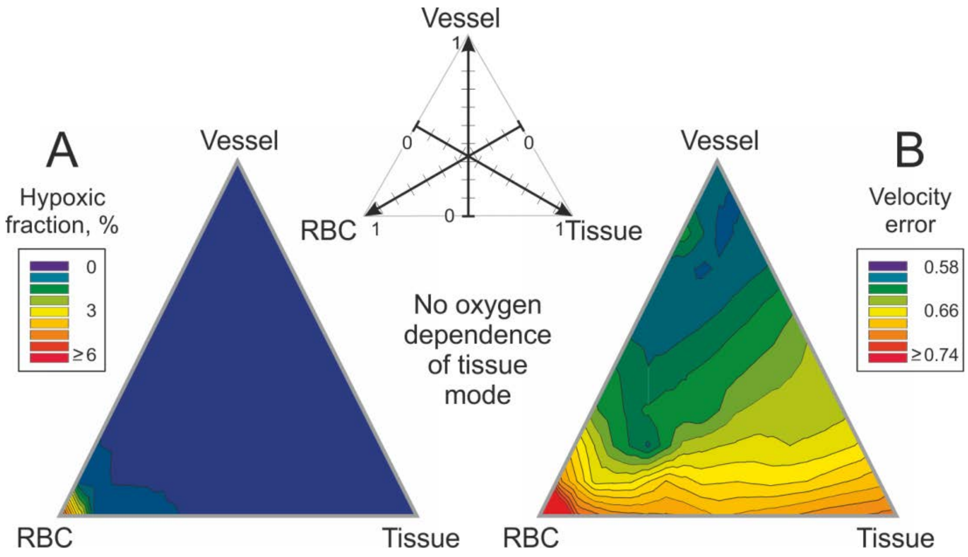

Figure S1. Effect of combining oxygen-sensitive vessel signaling and RBC signaling with oxygen-insensitive tissue signaling on tissue hypoxic fraction (A) and on velocity error (B). Figure design as in Figure 5. The resulting hypoxic fraction and V_{err} distributions deviate only minimally from those obtained assuming oxygen sensitivity of all three modes (Figure 5A,B).

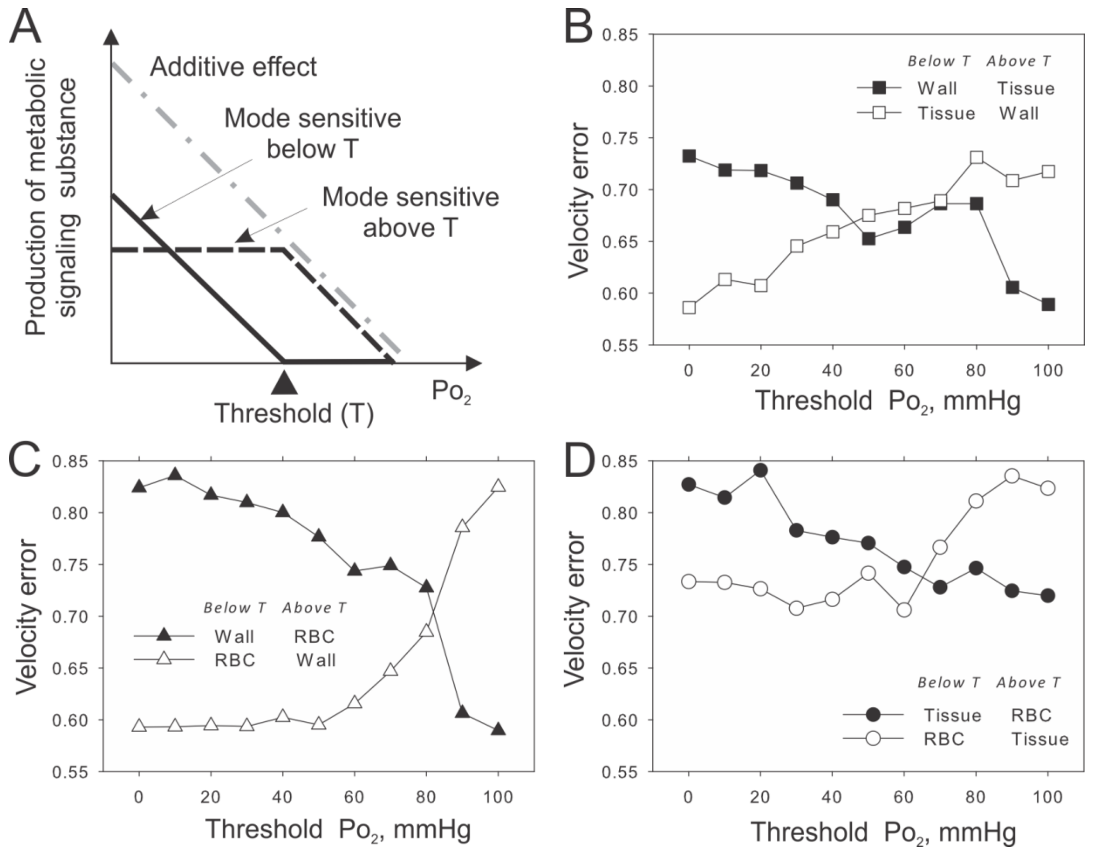

Figure S2. Results of simulations with combinations of two signaling modes, where one mode was assumed to exhibit PO_2 sensitivity in the high PO_2 range and the other in the low PO_2 range, with varying threshold values. A common reference PO_2 ($PO_{2\,ref}$) of 100 mmHg (instead of the individual $PO_{2\,ref}$ of 95 mmHg for vessel signaling, 115 mmHg for tissue signaling, and 92 mmHg, corresponding to a $SO_{2\,ref}$ of 0.93, for RBC signaling) was assumed for all three signaling modes. **A.** Scheme of assumed oxygen sensitivity for production of metabolic signaling substance. Oxygen sensitivity was adjusted so that one mode exhibits sensitivity above a certain threshold PO_2 (dashed line) and the other mode below this level (solid line), giving full-range oxygen sensitivity in combination (dashed/dotted line). Threshold PO_2 values were varied between 0 and 100 mmHg. **B-D.** Velocity errors resulting from combined action of two signaling modes assuming oxygen sensitivity characteristics according to (**A**). Combined action of wall signaling and tissue signaling (**B**), of wall signaling and RBC signaling (**C**), and of tissue signaling and RBC signaling (**D**). No combination exhibited a velocity error below that of vessel signaling alone. Much higher velocity errors were observed for all combinations not including vessel signaling. RBC signaling PO_2 sensitivity in low PO_2 ranges (<50 mmHg) scarcely changed V_{err} relative to vessel signaling alone and tissue signaling alone. These results show that low velocity errors are achieved only when wall signaling is included, with oxygen sensitivity in the upper range of PO_2 values.

Reference List

1. Pries AR, Secomb TW, Gessner T, Sperandio MB, Gross JF, Gaehtgens P. Resistance to blood flow in microvessels in vivo. *Circ Res* 1994;**75**:904-15.
2. Pries AR, Secomb TW, Gaehtgens P, Gross JF. Blood flow in microvascular networks - experiments and simulation. *Circ Res* 1990;**67**:826-34.
3. Reglin B, Secomb TW, Pries AR. Structural adaptation of microvessel diameters in response to metabolic stimuli: where are the oxygen sensors? *Am J Physiol Heart Circ Physiol* 2009;**297**:H2206-H2219.
4. Pries AR, Secomb TW, Gaehtgens P. Structural adaptation and stability of microvascular networks: theory and simulations. *Am J Physiol* 1998;**275**:H349-H360.
5. Pries AR, Reglin B, Secomb TW. Structural adaptation of microvascular networks: functional roles of adaptive responses. *Am J Physiol* 2001;**281**:H1015-H1025.
6. Pries AR, Secomb TW, Gaehtgens P. Design principles of vascular beds. *Circ Res* 1995;77:1017-23.
7. Jagger JE, Bateman RM, Ellsworth ML, Ellis CG. Role of erythrocyte in regulating local O2 delivery mediated by hemoglobin oxygenation. *Am J Physiol* 2001;**280**:H2833-H2839.
8. Segal SS, Duling BR. Flow control among microvessels coordinated by intercellular conduction. *Science* 1986;**234**:868-70.
9. Pries AR, Reglin B, Secomb TW. Structural response of microcirculatory networks to changes in demand: information transfer by shear stress. *Am J Physiol* 2003;284:H2204-H2212.
10. Collins DM, McCullough WT, Ellsworth ML. Conducted vascular responses: communication across the capillary bed. *Microvasc Res* 1998;**56**:43-53.
